# Development and validation of a scale on the difficulties of AI technology integration in teaching for primary and secondary school teachers

**DOI:** 10.3389/fpsyg.2026.1902647

**Published:** 2026-08-04

**Authors:** Shoujun Qiao, Yong Nie, Fuhe Niu, Zhenfeng He, Longlong Chen

**Affiliations:** 1 Faculty of Education, Shaanxi Normal University, Xi'an, China; 2 English Teaching Group, Central School of Zhouji, Huoqiu, China; 3 School of Teacher Development, Shaanxi Normal University, Xi'an, China

**Keywords:** cognition-emotion-volition-behavior framework, difficulties in AI technology integration in teaching, measurement invariance, primary and secondary school teachers, scale development

## Abstract

The lack of a comprehensive, theoretically grounded measurement instrument has constrained the systematic diagnosis of multidimensional barriers faced by K−12 teachers in integrating artificial intelligence (AI) into instruction. To address this gap, this study developed and validated a multidimensional scale based on the Cognitive–Emotional–Volitional–Behavioral (CEVB) heuristic framework. Through semi-structured interviews, expert consultation, and pilot testing, an initial 20-item pool was formed and administered to 546 teachers from central and western Anhui Province, China. Psychometric evaluation encompassed item analysis, exploratory factor analysis (EFA), confirmatory factor analysis (CFA), reliability testing, and validity and measurement invariance assessments. Item analysis confirmed satisfactory discrimination and differentiating power. EFA extracted a clear four-factor structure—cognitive understanding difficulties, affective attitudinal difficulties, conative motivational difficulties, and behavioral practice difficulties—accounting for 66.20% of the total variance. CFA supported both a first-order four-factor model (χ^2^/df = 2.014, CFI = 0.944, RMSEA = 0.061, SRMR = 0.056) and a higher-order model (χ^2^/df = 2.162, CFI = 0.935, RMSEA = 0.065, SRMR = 0.066). The scale demonstrated strong internal consistency (Cronbach's α = 0.915, McDonald's ω = 0.917). Convergent and discriminant validity were satisfactory, and strict measurement invariance was established across gender and school levels (primary, middle, and high school). Criterion-related validity was supported by significant positive correlations between scale dimensions and teacher AI literacy dimensions. Furthermore, structural equation modeling confirmed a significant sequential mediation pathway (F1 → F2 → F3 → F4), empirically validating the theoretical cascading process from cognitive deficits to affective resistance, then to motivational decline, and ultimately to behavioral impediments. This study provides a psychometrically robust, theory-driven diagnostic tool that enables phased and targeted interventions, moving beyond one-size-fits-all approaches to effectively support teachers' AI-integrated teaching.

## 1 Introduction

The rapid proliferation of artificial intelligence (AI) in education has brought transformative possibilities for personalized learning, intelligent assessment, and the optimisation of teaching resources (Wu et al., 2024; Zhou, 2024). Governments worldwide, particularly the Chinese government, have invested significantly in building AI-empowered educational ecosystems, with the expectation that teachers will effectively integrate AI tools into their daily instruction. However, despite sustained policy momentum and technological advances, a large body of evidence indicates that many primary and secondary school teachers face substantial difficulties when attempting to authentically incorporate AI into their teaching practice (Hu et al., 2024; Kazmaci et al., 2025). These difficulties are not merely technical; they are rooted in teachers' cognitive readiness, emotional responses, motivational states, and contextual implementation barriers. Consequently, understanding and measuring this multidimensional phenomenon is a prerequisite for designing effective, differentiated support strategies.

Existing research has explored various factors in teacher–AI interaction from different angles. Some studies, grounded in the Technology Acceptance Model, have focused on the effects of perceived usefulness and perceived ease of use on behavioral intention (Kong et al., 2024; Alejandro et al., 2024). Other studies have developed instruments to measure teacher AI literacy (Lim and Lee, 2023), AI self-efficacy (Chou et al., 2022, 2024), AI readiness (Ramazanoglu and Akin, 2024), and AI technostress (Kang et al., 2025). Although valuable, these scales typically capture isolated dimensions of difficulty and fail to systematically characterize the full chain of difficulties that teachers encounter—from understanding AI concepts to applying them in the classroom. More importantly, few studies have theorized the sequential and interdependent nature of teacher difficulties: cognitive gaps may trigger emotional resistance, which in turn weakens volitional motivation, ultimately hindering action. This limitation restricts the diagnostic precision of existing tools and impedes the development of holistic intervention frameworks.

To address this gap, the present study draws on the classic “Cognition-Emotion-Volition-Behavior” (CEVB) theoretical framework, which originates from educational psychology and behavior change theory (Guan and Hang, 2021; Xu, 2017). The CEVB framework posits that the transformation of human behavior follows a logical sequence: first cognitive understanding (cognition), then emotional experience (emotion), third volitional motivation (volition), and finally behavioral enactment (behavior). Transplanting this framework into the specific context of AI integration in teaching, we conceptualize teachers' difficulties in AI technology application as a multidimensional construct comprising four interrelated yet distinct dimensions: (1) difficulties in cognition and understanding—teachers lack sufficient conceptual and practical knowledge regarding the functions, pedagogical suitability, limitations, and ethical risks of AI tools; (2) difficulties in emotion and attitude—teachers experience negative emotions such as anxiety, frustration, a sense of helplessness due to the resource gap, and lack of trust when confronted with AI technologies; (3) difficulties in volition and motivation—teachers exhibit insufficient intrinsic drive, perceived imbalance between costs and benefits, and tendencies to avoid AI integration; and (4) difficulties in action and practice—external and internal barriers that impede actual classroom integration, including inadequate infrastructure, mismatches between tools and instructional contexts, lack of just-in-time technical support, and insufficient effectiveness of training.

By adopting this holistic, process-oriented perspective, the present study makes several theoretical and empirical contributions. First, it moves beyond fragmented single-dimension measurement and provides an integrated scale that captures the full landscape of teachers' difficulties in AI application. Second, by operationalising the CEVB framework within the domain of AI in education, it offers a theory-driven measurement tool suitable for cross-sectional and longitudinal studies. Third, the scale can serve as a diagnostic instrument for educational administrators and teacher trainers, enabling them to pinpoint precisely at which stage(s) teachers (or groups of teachers) encounter obstacles, and accordingly design tiered intervention strategies—for example, providing knowledge scaffolds for cognitive difficulties, offering emotional support for affective difficulties, adjusting incentive mechanisms for motivational difficulties, and improving infrastructure and technical support for action difficulties.

To clarify the theoretical status of the CEVB framework in this study, we emphasize that it serves primarily as a process-oriented analytical heuristic rather than a universal behavioral law. Its value in the specific context of AI integration lies in its sequential logic—positing that teachers' difficulties unfold along a temporal chain from cognition to emotion, then to volition, and finally to action. This stage-specific lens allows us to diagnose precisely where in this chain a given teacher encounters obstacles, rather than treating “difficulty” as a single, undifferentiated construct. Moreover, each of the four stages directly maps onto a distinct type of intervention lever: knowledge scaffolds for cognitive gaps, psychological support for emotional resistance, incentive adjustments for motivational deficits, and infrastructural improvements for action barriers. This one-to-one mapping between diagnostic dimension and practical remedy constitutes the specific added value of adopting CEVB for the purpose of scale development in this domain.

Therefore, the research question guiding this study is: Does the scale on primary and secondary school teachers' difficulties in AI technology integration in teaching, developed on the basis of the Cognition-Emotion-Volition-Behavior framework, exhibit sound factor structure, reliability, and validity? To answer this question, we generated an initial item pool through human–AI collaboration, revised the scale via expert consultation and pilot testing, and then evaluated its psychometric properties using a sample of 546 teachers from central and western China through exploratory factor analysis, confirmatory factor analysis, reliability testing, and assessments of convergent and discriminant validity.

## 2 Theoretical framework

The theoretical foundation of this scale is the “Cognition-Emotion-Volition-Behavior” (CEVB) framework, which has been widely influential in educational psychology and moral education research (Guan and Hang, 2021; Xu, 2017). The core proposition of this framework is that sustainable behavioral change—whether in learning, moral development, or professional practice—requires the sequential maturation of four psychological components: cognition (understanding what to do, why to do it, and how to do it), emotion (affective evaluation and feeling states toward the target behavior), volition (volitional drive, intention, and commitment), and behavior (actual execution and behavioral persistence). The absence or deficiency of any stage may disrupt the entire chain, resulting either in the failure to enact the behavior or in superficial enactment.

When this framework is applied to teachers' integration of AI technology into classroom instruction, the CEVB model provides a powerful explanatory mechanism. Teachers are not merely technology novices; they are situated professional practitioners whose decisions and behaviors are shaped by what they know, how they feel, what they value, and what the environment supports. Existing research has touched upon these dimensions from different angles, but few studies have modeled them as an interconnected system. Below we elaborate on each dimension, synthesize relevant literature, and justify its inclusion in the scale.

### 2.1 Dimension 1: difficulties in cognition and understanding

Difficulties in cognition and understanding refer to deficits, misconceptions, or insufficiencies in teachers' declarative and procedural knowledge regarding AI technology, including inadequate awareness of AI's pedagogical functions, operational requirements, and ethical-legal implications.

The first and most fundamental barrier is cognitive. Wang et al. (2023a) noted that teachers' AI readiness is significantly influenced by their conceptual understanding—that is, their awareness of AI's capabilities and boundaries. Without an accurate mental model, teachers cannot make informed decisions about which tool to choose, when to use it, and for what pedagogical purpose (Zhou, 2024). Empirical studies have identified several specific cognitive gaps: being unable to name more than two AI tools suitable for teaching (Lim and Lee, 2023); lacking clarity about how AI can address specific instructional problems such as differentiated instruction or real-time feedback (Guo et al., 2024); being unaware of local or school-level AI adoption policies (including funding and resource allocation); holding misconceptions about data privacy requirements and ethical risks (Viberg et al., 2024); and being unable to judge whether an AI tool matches their own curriculum standards, textbook versions, or students' prior knowledge (Kong et al., 2024). These cognitive deficits are not merely academic; they directly prevent teachers from moving from passive awareness to active experimentation. Therefore, in our framework, cognitive difficulties are conceptualized as the initial bottleneck. The scale assesses this dimension by measuring teachers' self-reported knowledge of specific AI tools, their understanding of AI's pedagogical functions, and their ability to access and interpret official guidelines.

### 2.2 Dimension 2: difficulties in emotion and attitude

Difficulties in emotion and attitude include negative affective states, evaluative beliefs, and attitudinal resistance that teachers experience when confronting AI technology, such as anxiety, fear of being replaced, a sense of helplessness due to resource gaps, and lack of trust.

Even when teachers possess adequate cognitive knowledge, their emotional reactions can still undermine subsequent motivation and action (Akçaba et al., 2025; Zhang et al., 2024). The emotional dimension is often underemphasised in technology acceptance models, yet it serves as a critical mediating variable. Synthesizing the existing literature, we identify six typical emotional difficulties: (1) operational anxiety—worry that AI tools are complicated to operate and may malfunction in real teaching situations; (2) loss of instructional control—fear that over-reliance on AI may erode one's professional autonomy (extending Carless and Boud, 2018; to the AI context); (3) relative deprivation—a sense of helplessness arising from comparing oneself to skilled AI-using teachers in developed regions (Hu et al., 2024); (4) frustration—intense negative emotions following unsuccessful initial attempts to use AI tools; (5) social contagion—colleagues' negative evaluations reducing one's own willingness to use AI; and (6) equity concerns—worry that students from low-income families may lack the necessary devices or internet access, thereby widening achievement gaps. These emotional difficulties are not merely individual traits; they are systematically shaped by institutional and socio-cultural contexts (Viberg et al., 2024). The present scale includes items capturing these diverse emotional reactions, with reverse coding such that higher scores indicate more severe emotional difficulties.

### 2.3 Dimension 3: difficulties in volition and motivation

Difficulties in volition and motivation refer to teachers' tendencies to delay, avoid, or discontinue the use of AI technology—even when cognitive and affective conditions are not unfavorable—due to insufficient intrinsic drive, lack of intentional commitment, and perceived imbalances in cost–benefit assessments.

The transition from emotion to action is mediated by volitional processes (Carless and Boud, 2018; DeKleijn, 2023). In the context of AI adoption, motivation is not a static trait but a dynamic state influenced by perceived value, perceived effort, and external incentives (Harsanti et al., 2025; Kong et al., 2024). Our framework identifies four key motivational difficulties: (1) lack of initiative—using AI only when mandated and failing to actively learn or experiment otherwise; (2) invisibility of evaluation—teachers perceive that their efforts to use AI are not recognized or rewarded in performance appraisals, leading to low persistence; (3) risk aversion—fear that making mistakes will disrupt classroom order or damage professional image; and (4) perceived time cost—believing that mastering AI tools consumes excessive lesson-preparation time with disproportionate benefits (Yao and Wang, 2024). These motivational difficulties are conceptually related to “perceived usefulness” and “perceived ease of use” in technology acceptance models, but they further emphasize the cost–benefit trade-offs in authentic teaching contexts. The scale assesses volitional and motivational difficulties through items measuring teachers' willingness to avoid AI, conditional compliance, and tendencies toward avoidance.

### 2.4 Dimension 4: difficulties in action and practice

Difficulties in action and practice refer to external and internal barriers that prevent teachers from actually implementing AI tools in real classroom settings, including infrastructural limitations, tool–context mismatches, gaps in technical support, ineffective training, and student-side constraints.

Ultimately, even when teachers possess adequate knowledge, positive emotions, and strong motivation, they may still be unable to implement AI-integrated teaching due to contextual constraints (Wu et al., 2024; Hu et al., 2024). This dimension is often the most visible but also the most complex, as it involves interactions among individual, organizational, and technological factors. Based on the existing literature and pilot testing, we identify five common action difficulties: (1) inadequate hardware—outdated computers, insufficient number of terminals, and poor network connectivity; (2) function–context mismatch—AI functions that do not correspond to actual classroom scenarios, leading to frequent disruptions; (3) lack of just-in-time technical support—no dedicated personnel to assist when breakdowns occur; (4) insufficient practical training—training that focuses primarily on theoretical explanations without sufficient hands-on classroom practice (Kazmaci et al., 2025); and (5) digital divide at the student end—inability to assign AI-based homework because students lack smartphones or internet access at home (Ramazanoglu and Akin, 2024). The scale measures action difficulties by asking teachers about their frequency of use, experienced failures, and access to support.

To summarize the rationale for adopting CEVB in this instrument development, three specific advantages are worth reiterating. First, it provides sequential diagnostic granularity: by partitioning the difficulty construct into four temporally ordered nodes, the framework enables researchers to pinpoint whether a teacher's primary bottleneck lies in conceptual understanding, affective appraisal, motivational commitment, or practical execution—rather than conflating all into a global score. Second, it permits the empirical examination of interdependent cascading effects: for instance, whether cognitive deficits exacerbate emotional anxiety, and whether emotional anxiety in turn undermines volitional drive. Such systemic mapping of inter-dimension relationships is unattainable when difficulties are measured as isolated variables. Third, and most importantly for the applied goal of this study, the framework yields actionable intervention correspondence: each dimension directly suggests a distinct remedial strategy. This diagnostic-to-intervention alignment is precisely what makes the CEVB framework practically useful for educational administrators and teacher trainers, beyond its theoretical appeal.

### 2.5 Theoretical integration and research hypotheses

The four dimensions described above are not independent of one another; rather, they form a causal chain: cognition → emotion → volition → behavior. This sequential assumption is consistent with the broader literature on behavioral change (Vygotsky, 1987; Carless, 2019) as well as the specific context of teacher professional learning. For example, incomplete knowledge breeds anxiety (emotion), which in turn reduces volitional drive (volition), ultimately translating into avoidance behavior (behavior). Moreover, even with strong motivation, action may be blocked by severe infrastructural barriers, highlighting the moderating role of contextual factors. By measuring all four dimensions simultaneously, the scale enables researchers and practitioners to identify the specific bottleneck (s) for a given teacher or school.

In summary, the theoretical framework of the Scale on Difficulties of AI Technology Integration in Teaching for Primary and Secondary School Teachers operationalises the CEVB model into four measurable factors: difficulties in cognition and understanding, difficulties in emotion and attitude, difficulties in volition and motivation, and difficulties in action and practice. This framework is empirically testable, educationally actionable, and grounded in a robust psychological tradition.

## 3 Methods

### 3.1 Participants

This study employed a combination of stratified random sampling and convenience sampling to survey primary and secondary school teachers in western Anhui Province from March to May 2026. To ensure sample representativeness, the sampling strategy comprehensively considered variations in region (urban, county-seat, rural), school level (elementary, middle, high), and subject area. Data were collected through an online survey using the “Questionnaire Star” platform. During the survey process, this study strictly adhered to ethical guidelines: an informed consent section was placed on the first page of the questionnaire, clearly stating the research purpose, the principles of anonymity and confidentiality, and the right to withdraw at any time without penalty. Teachers could proceed to answer the questions only after voluntarily providing consent. The questionnaire link and QR code were distributed via snowball sampling through school WeChat work groups.

To ensure data quality, the questionnaire included embedded attention-check items and set a minimum response time limit of 150 s. After data collection, invalid responses—those with insufficient response time, failure on attention-check items, clearly patterned answers (e.g., consecutive selection of the same option), or missing critical information—were strictly removed. A total of 573 questionnaires were returned, yielding 546 valid responses, representing an effective response rate of 93.1%. The demographic composition of the valid sample was as follows: male teachers (*n* = 174, 31.9%), female teachers (*n* = 372, 68.1%); elementary school teachers (*n* = 131, 24.0%), middle school teachers (*n* = 298, 54.6%), high school teachers (*n* = 117, 21.4%).

### 3.2 Instruments

#### 3.2.1 Scale on difficulties of AI technology integration in teaching for primary and secondary school teachers

The development of this scale was grounded in the core conceptualization and existing literature, drawing on the “Cognition-Emotion-Volition-Behavior” (CEVB) framework to initially construct four dimensions of difficulty: cognition and understanding, emotion and attitude, volition and motivation, and action and practice. To align with the practical realities of primary and secondary school teachers, the study employed convenience sampling to conduct semi-structured interviews with 10 teachers (5 male, 5 female) in western Anhui Province, exploring their authentic experiences and coping strategies in AI teaching applications. Based on these interviews, an initial pool of 24 items was generated. Subsequently, five educational technology experts and lead teachers assessed the content relevance and clarity of the items, resulting in the removal of four items with weak relevance or ambiguous wording. A pre-test questionnaire comprising 20 items was thus formed. The scale used a Likert 5-point response format (1 = strongly disagree, 5 = strongly agree), with items 7 through 20 reverse-scored. Higher total scores on each dimension and on the overall scale indicated more severe difficulties faced by teachers in integrating AI into teaching.

#### 3.2.2 Criterion Instrument

To examine the criterion-related validity of the Scale on Difficulties of AI Technology Integration in Teaching for Primary and Secondary School Teachers, this study adopted the AI Literacy Scale (AILS) developed by Wang et al. (2023b) as the criterion instrument. The rationale for selecting this scale is that AI literacy is theoretically closely related to difficulties in AI technology integration: the lower a teacher's AI literacy level, the greater the cognitive, emotional, motivational, and action difficulties they are likely to encounter in the process of teaching integration. Therefore, the two constructs should exhibit a significant negative correlation.

The AILS is constructed based on a four-dimensional framework of “awareness, usage, evaluation, and ethics” and comprises 12 items, using a Likert 7-point response format (1 = strongly disagree, 7 = strongly agree). Higher total scores on each dimension and on the overall scale indicate higher levels of teacher AI literacy (Wang et al., 2023a). In this study, the scale demonstrated a KMO value of 0.957 and a Cronbach's α of 0.959, with the Cronbach's α values for each dimension being 0.887, 0.906, 0.838, and 0.898 respectively, which indicates excellent reliability and validity of the scale. The complete item set is provided in Appendix A.

### 3.3 Statistical analysis

This study employed SPSS 25.0, Mplus 7.4, and AMOS 24.0 for data management and statistical analyses. Specifically, SPSS 25.0 was used to conduct item analysis, normality testing, internal consistency reliability, and criterion-related validity testing on the full sample, as well as exploratory factor analysis on one half of the data. Mplus 7.4 was applied to perform confirmatory factor analysis on the other half of the data, and to examine composite reliability, convergent validity, discriminant validity, and measurement invariance based on the full sample. AMOS 24.0 was utilized to carry out mediation effect analysis using the full sample (Wu, 2010).

We initially attempted to use WLSMV estimation with polychoric correlations given the ordinal nature of the data. However, this approach did not yield a stable solution for our measurement model. Following established recommendations, we therefore adopted the MLR estimator, which is appropriate for 5-point Likert-scale data with satisfactory normality and provides robust standard errors. All reported confirmatory results are based on MLR.

## 4 Results

### 4.1 Item analysis

This study conducted item analysis on the full sample, employing both the critical ratio (CR) method and the item-total correlation method to evaluate item discrimination. First, the total scale scores were ranked in descending order, and the upper and lower 27% of participants were assigned to the high-score and low-score groups, respectively. Independent-samples t–tests were then performed. The results (Table 1) showed that all items exhibited highly significant differences between the two groups (*t* > 3.000, *p* < 0.001), with Cohen's *d* values all exceeding 0.80, meeting the criterion for a large effect size, thus indicating satisfactory item discrimination. Moreover, the item-total correlation analyses revealed that each item was significantly positively correlated with the total scale score (based on 20 items), with correlation coefficients (*r1* and *r*_2_ > 0.400, *p* < 0.001), meeting the excellent standard of *r* ≥ 0.400, which further confirmed that all items possessed good discriminative power. In sum, these results consistently support that all items met the retention criteria. Therefore, no items were deleted in this study; all subsequent analyses—including exploratory factor analysis (EFA), confirmatory factor analysis (CFA), and reliability and validity testing—were conducted based on the full set of 20 retained items.

**Table 1:** Item analysis results of the scale (*n* = 546).

Item	High-score group (*M* ±*SD*)	Low-score group (*M* ±*SD*)	*t*	*d*	*r_1_*	*r_2_*
A1	3.32 ± 1.198	1.94 ± 0.817	11.919^***^	1.348	0.586^***^	0.513^***^
A2	3.35 ± 1.154	2.03 ± 0.762	11.978^***^	1.355	0.607^***^	0.523^***^
A3	3.96 ± 0.960	2.47 ± 1.038	13.161^***^	1.488	0.594^***^	0.558^***^
A4	3.48 ± 1.066	2.05 ± 0.785	13.473^***^	1.524	0.623^***^	0.560^***^
A5	3.29 ± 1.149	1.98 ± 0.740	11.941^***^	1.351	0.622^***^	0.543^***^
A6	3.53 ± 1.06	2.11 ± 0.792	13.422^***^	1.518	0.642^***^	0.569^***^
A7	3.24 ± 1.256	2.05 ± 0.935	9.455^***^	1.069	0.427^***^	0.414^***^
A8	3.41 ± 1.198	2.08 ± 0.876	11.209^***^	1.268	0.482^***^	0.469^***^
A9	3.31 ± 1.107	1.90 ± 0.813	12.820^***^	1.450	0.584^***^	0.557^***^
A10	3.62 ± 1.009	2.04 ± 0.798	15.353^***^	1.736	0.660^***^	0.622^***^
A11	3.37 ± 1.123	1.82 ± 0.695	14.663^***^	1.659	0.661^***^	0.620^***^
A12	3.80 ± 1.067	2.12 ± 0.950	14.717^***^	1.664	0.631^***^	0.591^**^
A13	3.75 ± 0.931	2.00 ± 0.779	18.044^***^	2.040	0.706^***^	0.676^***^
A14	3.74 ± 0.833	1.94 ± 0.781	19.681^***^	2.225	0.702^***^	0.690^***^
A15	3.94 ± 0.992	2.24 ± 0.971	15.315^***^	1.731	0.633^***^	0.609^***^
A16	3.70 ± 0.977	2.06 ± 0.889	15.557^***^	1.759	0.619^***^	0.596^***^
A17	3.68 ± 0.826	2.09 ± 0.838	16.860^***^	1.906	0.661^***^	0.645^***^
A18	3.45 ± 0.990	1.79 ± 0.707	17.036^***^	1.927	0.666^***^	0.630^***^
A19	3.42 ± 1.110	1.85 ± 0.769	14.516^***^	1.642	0.635^***^	0.584^***^
A20	3.54 ± 1.041	1.78 ± 0.829	16.533^***^	1.870	0.645^***^	0.641^***^

^***^p < 0.001. *r*_1_ denotes the Pearson correlation coefficient between each item and the total scale score; *r*_2_ denotes the Spearman correlation coefficient between each item and the total scale score.

### 4.2 Normality test

For cross-validation purposes, the total sample data were randomly and equally split into two halves: one half was used for exploratory factor analysis (EFA), and the other for confirmatory factor analysis (CFA). To inform the appropriate selection of subsequent data processing methods, normality tests were performed separately on these two subsamples using SPSS 25.0, based on indicators such as skewness and kurtosis (see Table 2).

**Table 2:** Normality test of the survey data for the scale.

Item	First half of the sample (*N* = 273)					Second half of the sample (*N* = 273)				
	M	SD	Sk	Ku	Nor	M	SD	Sk	Ku	Nor
A1	2.44	1.080	0.387	−0.523	Y	2.56	1.143	0.556	−0.390	Y
A2	2.51	1.008	0.508	−0.141	Y	2.70	1.060	0.483	−0.284	Y
A3	3.16	1.144	−0.016	−0.821	Y	3.35	1.137	−0.020	−1.057	Y
A4	2.61	1.072	0.464	−0.395	Y	2.81	1.035	0.401	−0.581	Y
A5	2.46	1.018	0.389	−0.294	Y	2.62	1.040	0.577	−0.096	Y
A6	2.75	1.019	0.235	−0.492	Y	2.77	1.030	0.394	−0.289	Y
A7	2.65	1.102	0.333	−0.535	Y	2.78	1.196	0.302	−0.776	Y
A8	2.68	1.062	0.268	−0.395	Y	2.90	1.154	0.238	−0.723	Y
A9	2.46	1.046	0.423	−0.371	Y	2.67	1.078	0.413	−0.431	Y
A10	2.79	1.089	0.295	−0.562	Y	2.87	1.030	0.361	−0.404	Y
A11	2.49	1.036	0.434	−0.273	Y	2.67	1.065	0.579	−0.230	Y
A12	2.90	1.157	0.080	−0.773	Y	3.03	1.180	0.071	−0.962	Y
A13	2.84	1.085	0.335	−0.485	Y	2.85	1.034	0.365	−0.433	Y
A14	2.84	1.083	0.202	−0.614	Y	2.84	1.044	0.183	−0.619	Y
A15	3.07	1.153	−0.093	−0.756	Y	3.16	1.088	0.079	−0.817	Y
A16	2.82	1.197	0.240	−0.843	Y	2.89	1.071	0.250	−0.653	Y
A17	2.85	0.997	0.208	−0.436	Y	2.90	0.998	0.118	−0.469	Y
A18	2.50	1.033	0.449	−0.356	Y	2.67	1.062	0.420	−0.519	Y
A19	2.40	1.090	0.553	−0.347	Y	2.78	1.079	0.376	−0.535	Y
A20	2.48	1.151	0.502	−0.472	Y	2.83	1.093	0.262	−0.719	Y

### 4.4 Exploratory factor analysis

Exploratory factor analysis (EFA) was conducted on one half of the data using SPSS 25.0. The results from the first half of the sample (*n* = 273) yielded a *KMO* value of 0.893 and a significant Bartlett's test of sphericity (χ^2^ = 3,080.055, *p* < 0.001), indicating satisfactory partial correlations among items and confirming the suitability of the data for factor analysis. Principal axis factoring was employed to extract common factors, followed by orthogonal rotation using the varimax method. Item deletion was conducted progressively based on the following criteria: (a) factor loading less than 0.45; (b) measure of sampling adequacy (*MSA*) for an item below 0.80; (c) substantial cross-loadings; and (d) item assignment inconsistent with the theoretical construct. Additionally, retained factors were required to have eigenvalues greater than 1 and to contain at least three items each. As shown in Table 3, the factor-item structure derived from the EFA largely corresponded to the dimension-item structure prespecified in the scale development, and therefore no items were deleted and the original factor labels were retained (Brandenburg, 2024). Although strict statistical thresholds were prespecified for item screening, all 20 items met the retention criteria in terms of factor loadings, MSA values, and theoretical congruence. Consequently, no items were removed, and the subsequent CFA and SEM analyses were conducted on the full 20-item scale.

**Table 3:** Exploratory factor analysis results of the scale (*n* = 273).

Item	Factor loading	*MSA*	Rotated factor loadings			
			1	2	3	4
A1	0.673	0.885^a^	**0.743**	0.219	0.062	0.059
A2	0.754	0.888^a^	**0.820**	0.196	0.027	0.078
A3	0.517	0.897^a^	**0.601**	0.163	0.026	0.170
A4	0.690	0.911^a^	**0.753**	0.227	0.067	0.064
A5	0.695	0.904^a^	**0.762**	0.175	0.112	0.092
A6	0.743	0.922^a^	**0.802**	0.186	0.103	0.110
A7	0.561	0.880^a^	0.050	0.006	0.191	**0.572**
A8	0.757	0.879^a^	0.076	−0.002	0.168	**0.815**
A9	0.527	0.931^a^	0.107	0.333	0.311	**0.473**
A10	0.626	0.914^a^	0.205	0.324	0.334	**0.557**
A11	0.595	0.891^a^	0.277	0.297	0.267	**0.544**
A12	0.673	0.900^a^	0.038	0.171	**0.641**	0.363
A13	0.723	0.901^a^	0.171	0.155	**0.671**	0.384
A14	0.742	0.910^a^	0.119	0.250	**0.729**	0.236
A15	0.777	0.860^a^	−0.014	0.246	**0.789**	0.181
A16	0.532	0.932^a^	0.290	**0.521**	0.273	0.004
A17	0.652	0.881^a^	0.252	**0.668**	0.155	0.136
A18	0.707	0.901^a^	0.169	**0.724**	0.306	0.071
A19	0.617	0.909^a^	0.278	**0.637**	0.192	0.102
A20	0.677	0.891^a^	0.271	**0.673**	0.056	0.171
Eigenvalue			7.556	2.971	1.641	1.072
Variance explained (%)			37.781	14.854	8.206	5.358
Cumulative variance explained (%)			37.781	52.634	60.840	66.198

The “Factor Loading” column refers to the initial (unrotated) loadings extracted via principal axis factoring; the four rightmost columns present the loadings after varimax rotation. The bold valuesăindicate the primary factor loadings of each item on its respective factor. These bolded loadings meet the retention criterion (factor loading ≥ 0.45) and represent the strongest association between each item and its theoretically assigned dimension in the exploratory factor analysis (EFA). The superscripts (if any) denote the factor dimension to which each item is theoretically assigned. They are used to distinguish the primary factor of each item from any cross-loadings, confirming that the item aligns with its intended subscale (Cognition, Emotion, Volition, or Action) based on the theoretical framework.

### 4.5 Confirmatory factor analysis

**Figure 1:**
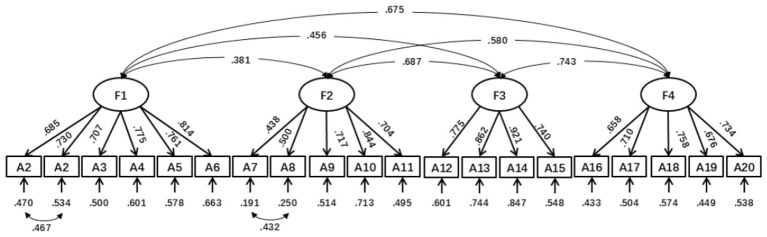
Confirmatory factor analysis results of the first-order measurement model of the scale (*n* = 273).

**Figure 2:**
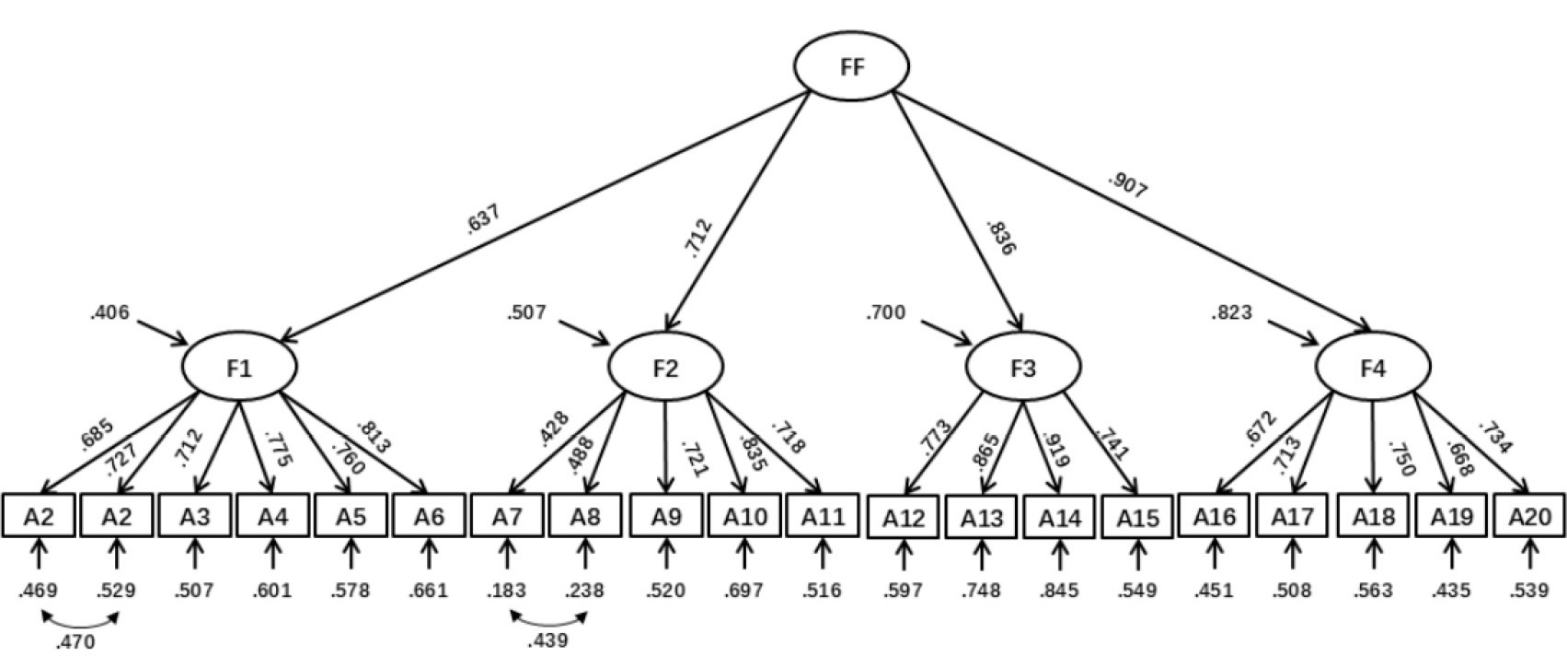
Confirmatory factor analysis results of the second-order measurement model of the scale (*n* = 273).

For the other half of the data, confirmatory factor analysis was conducted in this study. Mplus 7.4 was employed to establish first-order and second order measurement models (Figures 1, 2), and the MLR estimator was used for data analysis. The criteria for evaluating model goodness-of-fit were set as follows: a χ^2^*/df* ratio of less than 3 was considered excellent, and less than 5 was acceptable; both the *CFI* and *TLI* were required to exceed 0.90; for the *RMSEA* and *SRMR*, values below 0.05 were regarded as excellent, and below 0.08 as acceptable. If the fit indices did not meet the above standards, the model was revised based on modification indices (by allowing covariances among measurement error terms) or by deleting unsuitable items, until the model fit reached an acceptable level (Hooper et al., 2008; Hair et al., 2010).

Based on the modification indices, we sequentially established covariances between residual terms e7 and e8, and between e1 and e2, until the model fit indices reached acceptable levels (Table 4). The results showed that most fit indices for the revised first-order four factor model (Figure 1) and the second-order five factor model (Figure 2) reached excellent levels, with the χ^2^/df ratio achieving an excellent level, and both RMSEA and SRMR reaching acceptable levels. These findings indicate that the scale exhibits good construct validity, warranting the retention of all items, and is suitable for assessing the difficulties faced by primary and secondary school teachers in applying AI technology to teaching.

**Table 4:** Model fit indices for confirmatory factor analysis of the scale (*n* = 546).

Model	χ^2^	*df*	χ^2^ */df*	CFI	TLI	RMSEA(90% CI)	SRMR
First order	273.380	162	1.688	0.951	0.943	0.050 (0.040,0.060)	0.054
Second order	297.602	164	1.815	0.941	0.932	0.055 (0.045,0.064)	0.063

### 4.6 Composite reliability and convergent validity analysis

To further examine the validity of the scale, this study employed Mplus 7.4 with the MLR estimator to assess composite reliability, convergent validity, and discriminant validity based on the full sample data. To further examine the validity of the scale, this study used the full sample data to test its composite reliability, convergent validity, and discriminant validity. The evaluation criteria were as follows: item retention required that the item reliability (squared multiple correlations, *SMC*) fall between 0.40 and 0.95; composite reliability (*CR*) > 0.60 and average variance extracted (*AVE*) > 0.50 were taken as evidence of good convergent validity; additionally, discriminant validity was considered adequate if the square root of the *AVE* for each factor exceeded the absolute value of the inter-factor correlation coefficients.

The results presented in Table 5 show that, for both the first-order fourfactor model (Figure 1) and the second-order fourfactor model (Figure 2), the factor loadings of all items on their respective latent factors fell between 0.40 and 0.95, indicating satisfactory item reliability for each factor. The composite reliability (*CR*) values for all factors exceeded 0.60, demonstrating adequate composite reliability. The average variance extracted (*AVE*) values for all factors were greater than 0.50, indicating good convergent validity. Moreover, the correlation coefficients among most factors were smaller than the square roots of the corresponding *AVE* values, suggesting that the discriminant validity among the factors was satisfactory.

**Table 5:** Composite reliability, convergent validity, and discriminant validity of the scale (*n* = 546).

Modle	DIM	IR	CR	CV	DV			
		STD. LOADING	CR	AVE	*F1*	*F2*	*F3*	*F4*
First order	F1	0.680–0.828	0.892	0.581	**0.762**			
	F2	0.419–0.831	0.793	0.446	0.425	**0.668**		
	F3	0.758–0.870	0.884	0.656	0.383	0.707	**0.810**	
	F4	0.644–0.739	0.842	0.516	0.631	0.598	0.631	**0.718**
Second order	F1	0.681–0.830	0.892	0.580	**0.762**			
	F2	0.409–0.827	0.790	0.444	0.471	**0.666**		
	F3	0.753–0.873	0.884	0.656	0.481	0.627	**0.810**	
	F4	0.646–0.740	0.842	0.516	0.503	0.655	0.670	**0.719**

IR, item reliability; CR, composite reliability; CV, convergent validity; DV, discriminant validity; Bold diagonal values are the square roots of the AVE; values below the diagonal are Pearson correlation coefficients between factors. The same applies to subsequent tables.

### 4.7 Measurement invariance testing

To further evaluate the applicability of the scale, this study employed Mplus 7.4 with the MLR estimator to test measurement invariance via multi-group confirmatory factor analysis based on the full sample data. Specifically, we sequentially tested configural invariance (Model A), metric invariance (Model B), scalar invariance (Model C), and strict invariance (Model D) to assess the measurement equivalence of the Scale for Assessing the Difficulties of Primary and Secondary School Teachers in Applying AI Technology to Teaching across different gender groups and school levels (i.e., primary, junior high, and senior high). In the nested model comparisons, invariance was supported if Δ*CFI* < 0.010 and Δ*RMSEA* < 0.015, with Δ*TLI* < 0.010 and Δ*SRMR* < 0.030 used as supplementary criteria. As shown in Table 6, the scale demonstrated satisfactory measurement invariance both between male and female teachers and across the three school levels.

**Table 6:** Measurement invariance tests of the scale across gender and school level (*n* = 546).

Model	χ^2^	df	TLI	CFI	SMRM	RMSEA(90% CI)	MC	ΔCFI	ΔRMSEA
Invariance across gender									
A	495.136	324	0.963	0.957	0.049	0.044 (0.036,0.052)			
B	515.785	340	0.962	0.957	0.055	0.044 (0.036,0.051)	B vs. A	0.000	0.000
C	563.271	356	0.955	0.952	0.057	0.046 (0.039,0.053)	C vs. B	−0.005	0.002
D	565.683	376	0.959	0.958	0.057	0.043 (0.036,0.050)	D vs. C	0.006	−0.003
Invariance across school level (elementary, middle, high)									
A	753.235	486	0.945	0.936	0.057	0.055(0.047,0.063)			
B	794.106	518	0.943	0.937	0.063	0.054(0.047,0.061)	B vs. A	0.001	−0.001
C	854.221	550	0.937	0.935	0.064	0.055(0.048,0.062)	C vs. B	−0.002	0.001
D	895.334	590	0.937	0.939	0.067	0.053(0.046,0.060)	D vs. C	0.004	−0.002

MC = model comparison; Model A = configural invariance; Model B = metric invariance; Model C = scalar invariance; Model D = strict invariance.

### 4.8 Reliability testing

Based on the full sample data, the present study performed Cronbach's α reliability analysis using SPSS 25.0 and McDonald's ω reliability analysis using Mplus 7.4. Following the conventional psychometric criteria, reliability coefficients were evaluated as follows: > 0.900 indicated excellent, 0.800–0.899 good, and 0.700–0.799 acceptable. As presented in Table 7, the Cronbach's α coefficients for the subscales ranged from 0.802 to 0.895, with an overall α of 0.915 for the total scale; the McDonald's ω coefficients for the subscales ranged from 0.799 to 0.896, with an overall McDonald's ω of 0.917. All these reliability indices met the acceptable criterion of at least 0.700, indicating that the Scale for Assessing the Difficulties of Primary and Secondary School Teachers in Applying AI Technology to Teaching possesses good internal consistency reliability.

**Table 7:** Reliability analysis results of the scale (*n* = 546).

Factor	Item	Cronbach's α	Mcdonald's ω	Cronbach's α	Mcdonald's ω
F1	6	0.895	0.896	0.915	0.917
F2	5	0.802	0.799		
F3	4	0.881	0.882		
F4	5	0.838	0.840		

### 4.9 Criterion related validity analysis

Based on the full sample data, this study used SPSS 25.0 to conduct criterion-related validity testing. To further examine the criterion-related validity of the scale, this study conducted a criterion-related validity test using the full sample. As shown in Table 8, the four dimensions of the Scale for Assessing the Difficulties of Primary and Secondary School Teachers in Applying AI Technology to Teaching—namely, Difficulties in Cognition and Understanding, Difficulties in Emotion and Attitude, Difficulties in Volition and Motivation, and Difficulties in Action and Practice—were all significantly correlated with the subdimensions of AI literacy (i.e., Cognitive Recognition, Practical Application, Critical Evaluation, and Ethical Norms). These results indicate that the scale possesses good criterion-related validity.

**Table 8:** Criterion-related validity analysis results (*n* = 546).

	CI	PA	EA	EN
Difficulties in cognition and understanding	0.678^***^	0.689^***^	0.653^***^	0.676^***^
Difficulties in emotion and attitude	0.682^***^	0.678^***^	0.673^***^	0.690^***^
Difficulties in volition and motivation	0.713^***^	0.702^***^	0.702^***^	0.715^***^
Difficulties in action and practice	0.755^***^	0.761^***^	0.739^***^	0.752^***^

CI, cognitive identification; PA, practical application; EA, evaluation and assessment; EN, ethical norms. The symbol ^***^ indicates that the correlation coefficient is statistically significant at the *p* < 0.001 level (two-tailed). All dimensions of the developed scale show highly significant negative correlations with the subdimensions of the AI Literacy Scale (AILS), supporting the criterion-related validity of the instrument.

### 4.10 Testing the sequential mediation model using structural equation modeling

Based on the full sample data, this study used AMOS 24.0 to conduct mediation effect analysis. To further investigate the mechanism through which Difficulties in Cognition and Understanding (F1) affect Difficulties in action and practice (F4), this study employed the stepwise approach proposed by Baron and Kenny (1986) by sequentially incorporating Difficulties in emotion and attitude (F2) and Difficulties in volition and motivation (F3) into the model. Specifically, we established a no-mediation model (M1), two singlemediation models (M2 and M3), a chain mediation model (M4), and a multiple mediation model (M5) (see Table 9). The evaluation criteria for model goodness-of-fit were set as follows: 2.0 <χ^2^*/df* < 5.0, *CFI* > 0.90, *TLI* > 0.90, *RMSEA* < 0.08, and *SRMR* < 0.08. If these indices did not meet the criteria, model modifications were made based on modification indices. Additionally, the Bootstrap method with 2,000 resamples was used to generate 95% confidence intervals (*CI*); mediation effects were considered significant if the *CI* did not contain zero.

**Table 9:** Model fit indices, significance tests for mediation effects, and effect sizes based on the stepwise test approach (*n* = 546).

Model	*χ2* /df	CFI	TLI	RMSEA	SRMR	Pathway	*B*	95%CI [lower, upper]	*B%*
M1	2.284	0.982	0.977	0.049	0.026	F1 → F4	0.658	[0.560,0.726]	100
M2	2.251	0.971	0.964	0.048	0.036	F1 → F4	0.475	[0.372,0.574]	72.63
						F1 → F2 → F4	0.179	[0.125,0.251]	27.37
M3	2.621	0.969	0.962	0.055	0.039	F1 → F4	0.466	[0.375,0.564]	70.71
						F1 → F3 → F4	0.193	[0.138,0.265]	29.29
M4	2.517	0.957	0.950	0.053	0.045	F1 → F4	0.471	[0.382,0.566]	74.06
						F1 → F2 → F3 → F4	0.165	[0.119,0.224]	25.94
M5	2.487	0.959	0.951	0.052	0.043	F1 → F4	0.438	[0.346,0.535]	66.77
						F1 → F2 → F4	0.063	[0.007,0.134]	9.60
						F1 → F3 → F4	0.041	[0.003,0.094]	6.25
						F1 → F2 → F3 → F4	0.114	[0.074,0.175]	17.38

F1 = difficulties in cognition and understanding; F2 = difficulties in emotion and attitude; F3 = difficulties in volition and motivation; F4 = difficulties in action and practice. The B% column represents the proportion of the total effect accounted for by each specific pathway.

As shown in Table 9, all models exhibited good fit. In Model M5, Difficulties in cognition and understanding (F1) positively predicted Difficulties in emotion and attitude (F2), Difficulties in volition and motivation (F3), and Difficulties in action and practice (F4) (β = 0.425, *p* < 0.001; β = 0.101, *p* < 0.05; β = 0.421, *p* < 0.001, respectively); Difficulties in emotion and attitude (F2) positively predicted Difficulties in volition and motivation (F3) and Difficulties in action and practice (F4) (β = 0.144, *p* < 0.05; β = 0.663, *p* < 0.001, respectively); and Difficulties in volition and motivation (F3) positively predicted Difficulties in action and practice (F4) (β = 0.390, *p* < 0.001).

**Figure 3:**
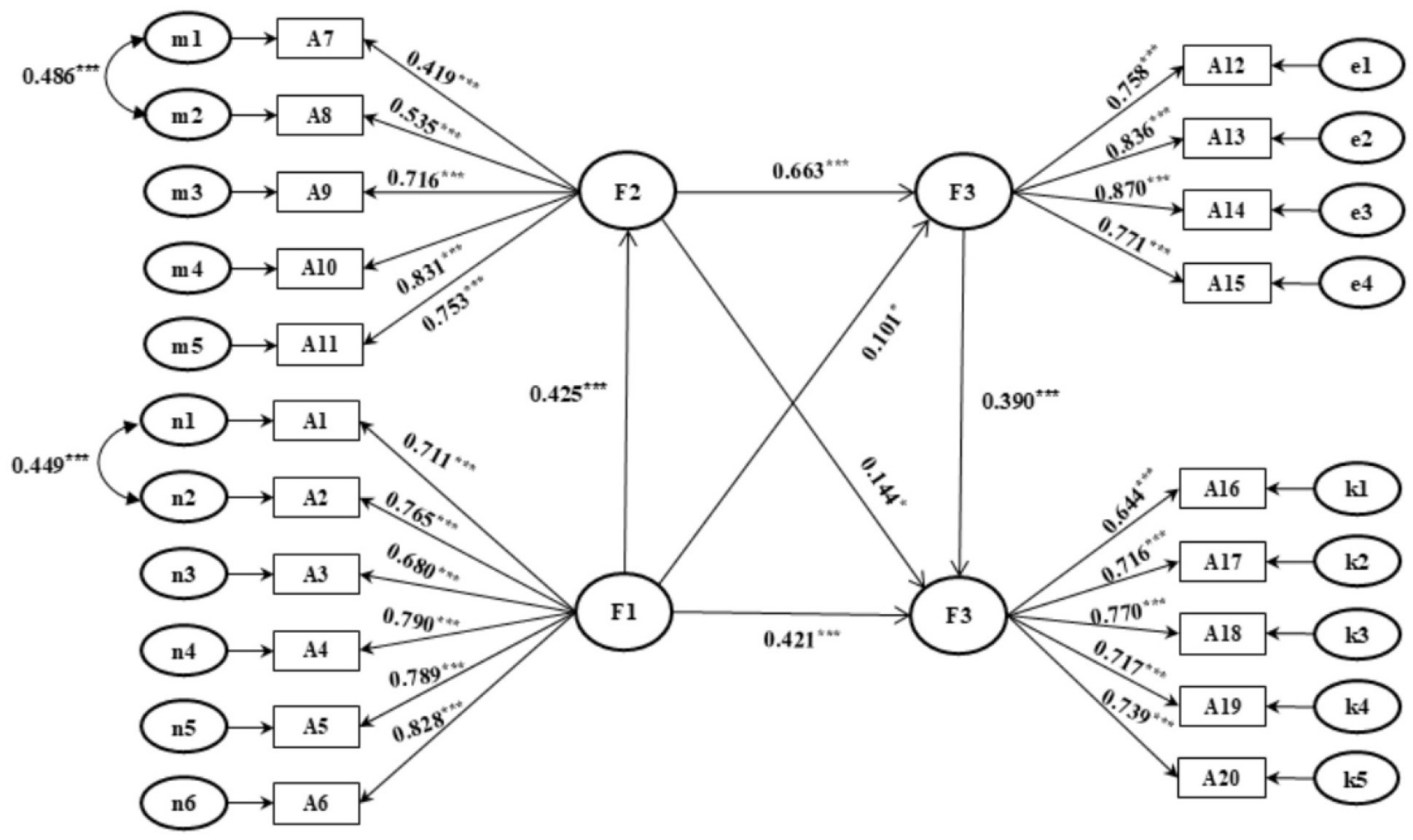
Multiple mediation effects of emotional and attitudinal difficulties (F2) and willingness and motivational difficulties (F3) on the relationship between cognitive and understanding difficulties (F1) and action and practice difficulties (F4) (*n* = 546). ***p < 0.001.

Bootstrap tests revealed that the direct effect of Difficulties in cognition and understanding (F1) on Difficulties in action and practice (F4) was significant (*B* = 0.438, *CI* [0.346, 0.535]), accounting for 66.77% of the total effect. The mediating effect of Difficulties in emotion and attitude (F2) between F1 and F4 was significant [*B* = 0.063, *CI* (0.007, 0.134)], with an effect size proportion of 9.60%; the mediating effect of Difficulties in volition and motivation (F3) between F1 and F4 was also significant [*B* = 0.041, *CI* (0.003, 0.094)], accounting for 6.25% of the total effect; and the chain mediating effect of F2 and F3 in sequence between F1 and F4 was significant (*B* = 0.114, *CI* [0.074, 0.175]), representing 17.38% of the total effect. Thus, Difficulties in emotion and attitude (F2) and Difficulties in volition and motivation (F3) play multiple mediating roles in the relationship between F1 and F4 (Figure 3).

## 5 Discussion and conclusion

Grounded in the classic the “Cognition-Emotion-Volition-Behavior” (CEVB) theoretical framework, this study developed the Scale for Assessing the Difficulties of Primary and Secondary School Teachers in Applying AI Technology to Teaching and subjected it to systematic psychometric evaluation using a sample of 546 primary and secondary school teachers. The findings not only confirmed the reliability and validity of the scale but also deepened the understanding of the internal structure of teachers' difficulties in AI application, offering practical guidance for educational practice.

First and foremost, the core conclusion of this study is that the difficulties faced by primary and secondary school teachers in applying AI technology to teaching constitute a multidimensional construct, comprising four distinct yet closely interrelated dimensions: Cognitive and Understanding Difficulties, Emotional and Attitudinal Difficulties, Difficulties in volition and motivation, and Action and Practice Difficulties. Moreover, this four-dimensional structure is governed by a single higher-order factor. This structure was consistently and robustly supported by both exploratory and confirmatory factor analyses. The data indicated that the second-order four-factor model exhibited good fit, suggesting that teachers' difficulties in AI application are not isolated obstacles but rather an integrated whole with inherent internal logic. This conclusion moves beyond previous fragmented perspectives that focused solely on technology acceptance (e.g., the TAM model) or a unitary conception of AI literacy, revealing that teachers' difficulties in AI application constitute a complete chain extending from internal psychological processing to externally observable behavioral impediments. The empirical distinction among the four dimensions corroborates the sequential nature of these difficulties: even when teachers possess a certain level of AI cognition (knowing), anxiety may still trigger emotional resistance (feeling); even in the absence of emotional aversion, perceived cost-benefit imbalances may undermine motivation (willing); and even with sufficient motivation, contextual constraints such as hardware limitations may derail implementation (acting). This provides a compelling theoretical explanation for common phenomena observed in educational settings, such as “high knowledge but low practice” or “willing heart but weak hands.”

Second, the Scale for Assessing the Difficulties of Primary and Secondary School Teachers in Applying AI Technology to Teaching demonstrates excellent psychometric properties. Item analysis indicated satisfactory discrimination for all items. The EFA extracted a clear four-factor structure, with a cumulative variance explained of 65.42%. The CFA results confirmed that both the first-order and second-order models achieved fit indices at excellent or acceptable levels (e.g., CFI > 0.95, RMSEA <0.08). The composite reliability (CR > 0.79) and internal consistency coefficients (α ranging from 0.737 to 0.895) indicated good reliability. Convergent and discriminant validity both met the established criteria, and strict measurement invariance was achieved across gender groups and school levels, demonstrating the measurement fairness and comparability of the scale across different populations. Furthermore, all dimensions of the scale were significantly correlated with the subdimensions of AI literacy, establishing sound criterion-related validity.

More importantly, our SEM-based chain mediation analysis offers the first empirical test of the sequential logic embedded in the CEVB framework within this domain. The significant indirect path through F2 and F3 (F1 → F2 → F3 → F4) confirms that difficulties do not operate as isolated parallel barriers; rather, cognitive deficits tend to trigger emotional resistance, which in turn dampens volitional motivation, and ultimately hampers classroom action. This cascading pattern explains why many interventions that target only one aspect (e.g., providing knowledge training) often fail to produce sustained behavioral change – they ignore the downstream emotional and volitional bottlenecks. Thus, our study not only provides a validated measurement tool but also validates a heuristic process model that can guide future intervention design.

Finally, based on the four-dimensional diagnostic results of the scale, educational practice should shift from a “one-size-fits-all” approach to “precision intervention.” The suboptimal outcomes of many current AI teacher training programs are fundamentally attributable to a failure to address the specific root causes of difficulties. The present scale can help administrators precisely identify the bottlenecks of teachers' difficulties, thereby enabling tiered and targeted support. Specifically, for Cognitive and Understanding Difficulties, practical toolkits and curriculum-aligned case examples should be provided to build “scaffolding for knowledge;” for Emotional and Attitudinal Difficulties, a fault-tolerant mechanism and peer support network should be established to create a “psychological safety zone” that alleviates substitution anxiety and relative deprivation; for Willingness and Motivational Difficulties, AI application should be incorporated into visible performance evaluations, perceived time costs should be reduced, and “incentive mechanisms” should be recalibrated; and for Action and Practice Difficulties, it is imperative to improve infrastructure, provide on-site technical support and hands-on training, and strengthen the “ecological support system” to bridge the “last mile” from the training room to the real classroom.

## 6 Research implications

At the theoretical level, this study is the first to systematically introduce the “Cognition-Emotion-Volition-Behavior” (CEVB) framework into the domain of teachers' difficulties in AI technology integration in teaching, moving beyond the limitations of previous research that focused only on single dimensions. It confirms that the four difficulty dimensions are both distinct from one another and collectively subsumed under a higher-order overall difficulty construct. The differentiated pattern of inter-dimension correlations carries important theoretical implications: the correlation between emotion and volition was the highest (*r* = 0.707), directly supporting the sequential “emotion → volition” hypothesis; the correlation between cognition and action (*r* = 0.631) was higher than that between cognition and emotion, suggesting that cognitive deficits may more directly impede action than emotional factors. These findings provide a quantitative basis for future development of causal models along the “Cognition → Emotion → Volition → Behavior” chain.

At the practical level, the scale can serve as a diagnostic tool to help educational administrators accurately identify the primary types of difficulties faced by different teacher subgroups (e.g., by gender, school level, or region). For example, rural teachers may experience the double burden of both cognitive deficits and action constraints, requiring priority to be given to improving hardware and training provision. Based on diagnostic results, tiered intervention strategies can be designed: for cognition difficulties, focus on knowledge scaffolding and case-based training; for emotion difficulties, provide psychological support and peer assistance; for volition difficulties, adjust incentive mechanisms; for action difficulties, ensure hardware availability and technical support. Furthermore, the scale can be used to evaluate the actual effectiveness of teacher training programmes by comparing pre-test and post-test scores to quantify intervention outcomes.

## 7 Limitations and future research directions

This study has several limitations that should be acknowledged, along with corresponding avenues for future research.

First, limitations regarding sample representativeness. The sample was primarily drawn from the central and western regions of Anhui Province in China and did not cover the more developed eastern areas or other cultural backgrounds. Although the sample demonstrated good representativeness for this region, objective disparities in digital infrastructure and educational resources exist across regions. Future research should expand the sampling scope to conduct cross-regional and cross-cultural measurement invariance tests and establish normative data, thereby examining the scale's applicability across broader geographic areas and diverse cultural contexts.

Second, limitations regarding measurement methods. The scale relied on teacher self-reports, which may be susceptible to social desirability bias—particularly with respect to the dimension of “difficulties in volition and motivation,” where teachers might be reluctant to candidly disclose their avoidance tendencies. Additionally, although polychoric correlations are theoretically more appropriate for Likert-scale data, our exploratory factor analysis (EFA) used a Pearson correlation matrix. This choice was based on the 5-point response format with relatively balanced item distributions; simulation evidence has also shown that Pearson correlations can recover factor structure adequately under certain conditions. Moreover, the robustness of the structure was subsequently confirmed by confirmatory factor analysis (CFA) on an independent split-half sample (CFI = 0.944, RMSEA = 0.061). Nevertheless, future studies are encouraged to replicate the factor structure using polychoric correlations and robust estimation methods, and to integrate objective behavioral data such as classroom observations and system logs to triangulate self-report findings and further validate the scale's predictive power for actual “difficulties in action and practice.”

Third, limitations regarding causal inference and temporal stability. The cross-sectional design precludes assessment of temporal stability or dynamic changes in difficulties over time. Although the four-factor structure and higher-order model were empirically supported, the hypothesized causal sequential mechanism of “Cognition-Emotion-Volition-Behavior (CEVB)” was not directly tested. Future studies could employ longitudinal follow-up designs or cross-lagged panel models to examine test-retest reliability, track the transformation pathways and reciprocal interactions among the four types of difficulties over time, and thereby empirically validate the causal chain underlying the theoretical framework.

Fourth, limitations regarding conceptual purification. Although the criterion-related validity of the scale was statistically significant, some correlation coefficients were relatively high (e.g., the correlation between difficulties in action and practical AI application reached 0.761), suggesting a certain degree of conceptual overlap between AI application difficulties and AI literacy. Future research could further refine the item wording to accentuate the attributes of perceived resistance and contextual impediment inherent in difficulties, thereby enhancing the scale's independent explanatory power and discriminant validity.

Fifth, regarding the choice of estimator. Although the MLR estimator adopted in this study demonstrated good applicability under our data conditions and yielded robust model fit results, it should be acknowledged that WLSMV with polychoric correlations remains the theoretically preferable method for handling ordinal data. Since WLSMV did not converge to a stable solution in our dataset, we were unable to adopt it for the primary analyses. Future research is encouraged to apply WLSMV estimation with larger or differently structured samples to further cross-validate the factor structure reported in this study.

Despite these limitations, the scale demonstrates good reliability, validity, and measurement invariance, providing an effective tool for systematically diagnosing teachers' barriers to AI integration and laying an empirical foundation for subsequent theoretical development and intervention research.

## Data Availability

The raw data supporting the conclusions of this article will be made available by the authors, without undue reservation.

## Appendix

**Table A1:**

Factor name	Item
Cognition and understanding	1. I can accurately name three or more AI tools suitable for educational settings.
	2. I understand the specific challenges AI technology can address in education as well as its limitations.
	3. I am fully aware of the local government's policy support for AI-powered education applications.
	4. I can obtain the usage guide for AI tools through official channels.
	5. I understand the purposes of AI tools in collecting student data and the relevant requirements for student privacy protection.
	6. I can determine whether an AI tool aligns with the textbook version and students' learning foundation.
Emotion and attitude	7. When considering the use of AI technology in teaching, I feel anxious due to concerns about its complexity.
	8. I'm concerned that over-reliance on AI technology could undermine my teaching autonomy.
	9. I feel powerless due to the resource gap when see teachers in some developed regions using AI technology skillfully.
	10. I feel really frustrated when the AI tool doesn't work well.
	11. I'm concerned that students may struggle to use AI tools due to financial constraints, which could compromise the effectiveness of their learning.
Volition and motivation	12. I probably wouldn't have taken the initiative to learn how to use AI tools if schools didn't make it mandatory.
	13. I'm concerned that the effectiveness of AI tools won't be reflected in teaching evaluations, therefore I lack the motivation to keep using AI tools.
	14. I was hesitant to use AI tools, fearing that operational errors might disrupt the class progress.
	15. I think that learning AI technology would consume a significant amount of lesson preparation time, this would reduce my willingness to adopt it.
Action and practice	16. I was forced to abandon AI tools due to insufficient school facilities.
	17. I often stop using AI tools when they fail to align with my teaching needs.
	18. I cannot find effective solutions (e.g., lack of dedicated technical support) when AI tools experience technical failures.
	19. The AI teaching training I attended was mostly theoretical lectures, lacking hands-on practice for classroom application.
	20. I cannot provide extended after-class activities because students' families lack devices with AI tools.

## References

[B1] Akçaba P. Toklu D. A. Akcil U. (2025). Examining the attitudes and anxiety of teachers and administrators toward artificial intelligence: relational browsing. BRAIN Broad Res. Artif. Intell. Neurosci. 15, 325–337. doi: 10.70594/brain/15.4/22

[B2] Alejandro I. M. V. Sanchez J. M. P. Sumalinog G. G. Mananay J. A. Goles C. E. Fernandez C. B. (2024). Pre-service teachers‘technology acceptance of artificial intelligence (AI) applications in education. STEM Educ. 4, 445–465. doi: 10.3934/steme.2024024

[B3] Baron R. M. Kenny D. A. (1986). The moderator–mediator variable distinction in social psychological research: Conceptual, strategic, and statistical considerations. J. Pers. Soc. Psychol. 51, 1173–1182. doi: 10.1037/0022-3514.51.6.1173 3806354

[B4] Brandenburg N. (2024). Factor retention in ordered categorical variables: benefits and costs of polychoric correlations in eigenvalue-based testing. Behav. Res. Methods 56, 7241–7260. doi: 10.3758/s13428-024-02417-0 38710985 PMC11362475

[B5] Carless D. (2019). Feedback loops and the longer-term: toward feedback spirals. Assess. Eval. High. Educ. 44, 705–714. doi: 10.1080/02602938.2018.1531108

[B6] Carless D. Boud D. (2018). The development of student feedback literacy: enabling uptake of feedback. Assess. Eval. High. Educ. 43, 1315–1325. doi: 10.1080/02602938.2018.1463354

[B7] Chou C.-M. Shen T.-C. Shen T.-C. Shen C.-H. (2022). The level of perceived efficacy from teachers to access AI-based teaching applications. Res. Pract. Technol. Enhanc. Learn. 18:021. doi: 10.58459/rptel.2023.18021

[B8] Chou C.-M. Shen T.-C. Shen T.-C. Shen C.-H. (2024). Developing and validating an AI-supported teaching applications‘self-efficacy scale. Res. Pract. Technol. Enhanc. Learn. 19:035. doi: 10.58459/rptel.2024.19035

[B9] DeKleijn R. A. M. (2023). Supporting student and teacher feedback literacy: an instructional model for student feedback processes. Assess. Eval. High. Educ. 48, 186–199. doi: 10.1080/02602938.2021.1967283

[B10] Guan J. Hang N. (2021). Zhi qing yi xing: si wei yi ti zhu lao zhong hua min zu gong tong ti yi shi [cognition-emotion-volition-behavior: four-in-one to forge the sense of community of the Chinese nation]. J. Nankai Univ. 6, 53–67.

[B11] Guo S. Shi L. Zhai X. (2024). Validating an instrument for teachers‘acceptance of artificial intelligence in education (Version 1). arXiv. doi: 10.1007/s10639-025-13338-6

[B12] Hair J. F. Black W. C. Babin B. J. Anderson R. E. (2010). Multivariate Data Analysis: A Global Perspective, 7th Edn. Upper Saddle River, NJ: Pearson Education International.

[B13] Harsanti H. R. Sudibjo N. Riady S. Yu P. (2025). Exploring Indonesian teachers‘intention to use artificial intelligence in schools: the impact of digital leadership, digital readiness, and perceived usefulness. Cogent Soc. Sci. 11. doi: 10.1080/23311886.2025.2593596

[B14] Hooper D. Coughlan J. Mullen M. R. (2008). Structural equation modelling: guidelines for determining model fit. Electron. J. Bus. Res. Methods 6, 53–60.

[B15] Hu X. Sui H. Geng X. Zhao L. (2024). Constructing a teacher portrait for the artificial intelligence age based on the micro ecological system theory: a systematic review. Educ. Inf. Technol. 29, 16679–16715. doi: 10.1007/s10639-024-12513-5

[B16] Kang X. Nie Y. Lyu P. . (2025). Sheng cheng shi ren gong zhi neng bo lang xia gao xiao wai yu jiao shi ji shu ya li liang biao kai fa yu jian yan [development and validation of a technostress scale for foreign language teachers under the wave of generative artificial intelligence]. Digit. Educ. 11, 39–46.

[B17] Kazmaci A. Cek K. Altinay F. Altinay Z. Dagli G. (2025). Influence of theoretical and practical artificial intelligence knowledge on the primary school teachers' sustainable AI integration ability: mediating effects of beliefs and attitudes. Front. Psychol. 16:1628557. doi: 10.3389/fpsyg.2025.1628557 40837268 PMC12361219

[B18] Kong S. C. Yang Y. Hou C. (2024). Examining teachers‘behavioural intention of using generative artificial intelligence tools for teaching and learning based on the extended technology acceptance model. Comput. Educ. Artif. Intell. 7:100328. doi: 10.1016/j.caeai.2024.100328

[B19] Lim H. Lee M. (2023). Development and validation of the pre-secondary school teacher's artificial intelligence literacy scale. Korean Assoc. Learner-Cent. Curric. Instr. 23, 875–892. doi: 10.22251/jlcci.2023.23.12.875

[B20] Ramazanoglu M. Akin T. (2024). AI readiness scale for teachers: development and validation. Educ. Inf. Technol. 30, 6869–6897. doi: 10.1007/s10639-024-13087-y

[B21] Viberg O. Cukurova M. Feldman-Maggor Y. Alexandron G. Shirai S. Kanemune S. . (2024). What explains teachers‘trust in AI in education across six countries? Int. J. Artif. Intell. Educ. 35, 1288–1316. doi: 10.1007/s40593-024-00433-x

[B22] Vygotsky L. S. (1987). The Collected Works of L. S. Vygotsky: Vol. 1. Problems of General Psychology. New York, NY: Plenum Press.

[B23] Wang B. Rau P.-L. P. Yuan T. (2023a). Measuring user competence in using artificial intelligence: validity and reliability of artificial intelligence literacy scale. Behav. Inf. Technol. 42, 1324–1337. doi: 10.1080/0144929X.2022.2072768

[B24] Wang X. Li L. Tan S. C. Yang L. Lei J. (2023b). Preparing for AI-enhanced education: conceptualizing and empirically examining teachers‘AI readiness. Comput. Human Behav. 146:107798. doi: 10.1016/j.chb.2023.107798

[B25] Wu D. Zhang X. Wang K. Wu L. Yang W. (2024). A multi-level factors model affecting teachers' behavioral intention in AI-enabled education ecosystem. Educ. Technol. Res. Dev. 73, 135–167. doi: 10.1007/s11423-024-10419-0

[B26] Wu M. (2010). Jie gou fang cheng smo xing AMOS de cao zuo yu ying yong [Operation and application of structural equation modeling with AMOS], 2nd Edn. Chongqing: Chongqing University Press.

[B27] Xu S. (2017). Yang cheng jiao yu “si wei yi ti” de li lun qian ti—ji yu zhi, qing, yi, xing de gai nian qu fen [the theoretical premise of the “four-in-one” nurturing education: based on the conceptual distinction of cognition, emotion, volition, and behavior]. Univ. Educ. 11, 24–28.

[B28] Yao N. Wang Q. (2024). RETRACTED: Factors influencing pre-service special education teachers‘intention toward AI in education: digital literacy, teacher self-efficacy, perceived ease of use, and perceived usefulness. Heliyon 10:e34894. doi: 10.1016/j.heliyon.2024.e34894 39149079 PMC11325385

[B29] Zhang C. Hu M. Wu W. Kamran F. Wang X. (2024). Unpacking perceived risks and AI trust influences pre-service teachers' AI acceptance: a structural equation modeling-based multi-group analysis. Educ. Inf. Technol. 30, 2645–2672. doi: 10.1007/s10639-024-12905-7

[B30] Zhou C. (2024). Teacher's perceptions of applying artificial intelligence in education: a systematic review. World J. Soc. Sci. Res. 11:p62. doi: 10.22158/wjssr.v11n3p62

